# Follicular Thyroid Cancer Metastasis to the Urinary Bladder: Report of a Case and Review of the Literature

**DOI:** 10.1155/2012/178915

**Published:** 2012-08-01

**Authors:** N. Grivas, Z. Housianitis, M. Doukas, N. E. Stavropoulos

**Affiliations:** ^1^Department of Urology, G. Hatzikosta General Hospital, Makriyianni Avenue, Perfecture of Epirus, 45001 Ioannina, Greece; ^2^Department of Pathology, G. Hatzikosta General Hospital, Makriyianni Avenue, Perfecture of Epirus, 45001 Ioannina, Greece

## Abstract

Thyroid cancer metastasis to the urinary bladder is a very rear condition. To the authors' knowledge there have been only 2 cases reported in the literature. Herein a case is reported of a metastatic bladder tumor in a 73-year-old woman with history of thyroid and breast cancer. Gross hematuria was the initial symptom of her metastatic disease. Pathology of the resected mass revealed a follicular thyroid cancer metastasis. This case illustrates that follicular carcinoma of the thyroid may have a variable presentation, including hematuria.

## 1. Introduction

Metastatic disease to the urinary bladder contributes to less than 1% of all bladder neoplasms [1]. Soft tissue metastasis from follicular thyroid cancer is seen most commonly in the lungs and occasionally in the liver and kidneys. We report a case of a woman with a history of breast and thyroid cancer presenting with hematuria as first manifestation of metastatic disease.

## 2. Case Presentation

An 73-year-old female patient presented to our hospital with a chief complaint of painless macroscopic hematuria. She had a past medical history of follicular thyroid cancer status after thyroidectomy 5 months ago, breast cancer status after right mastectomy 17 years ago, hypertension, and osteoporosis. She was taking thyroxine 100 mg, omeprazole 20 mg, domperidone 20 mg, and perindopril/amlodipine 5 mg/5 mg each in once daily dose. 

The patient reported no prior episodes of gross hematuria. She had no history of smoking or occupational/chemical exposure. She denied any history of nephrolithiasis or urinary tract infections. On physical examination she had no flank pain or fever and she voided wine-colored urine. Blood tests including hemoglobin, hematocrit, liver function tests; and coagulation studies were within normal limits. Her urinalysis revealed RBCs >200 and no WBCs. An ultrasound was obtained and revealed a suspicious mass along the right lateral wall of the bladder. The kidneys were normal in appearance. 

**Figure 1:**
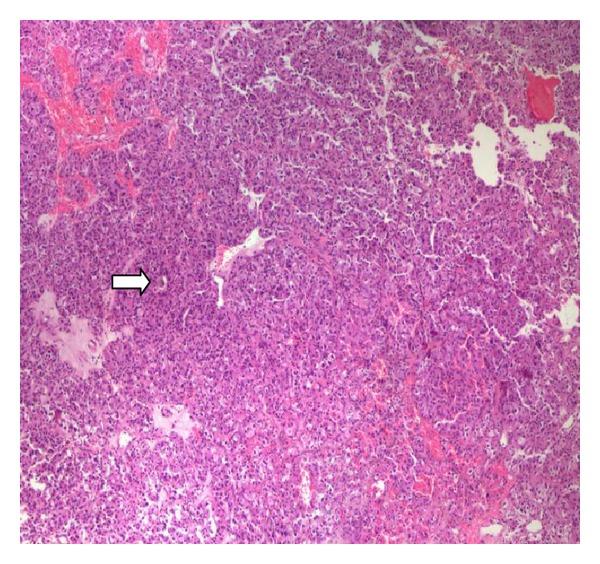
Biopsy material consisted of neoplastic cells arranged in cords (H-EX4). Focal presence of hemosiderin deposition (arrow).

**Figure 2:**
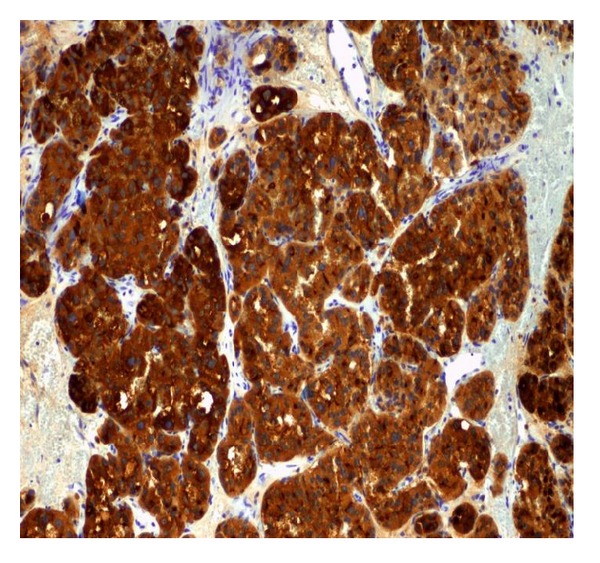
Immunohistochemical positivity for Thyroglobulin.

Differential diagnosis included primary transitional cell carcinoma. However, her previous thyroid and breast cancer history could make this an unusual presentation of metastatic carcinoma to the bladder. We decided to proceed with cystoscopy, and transurethral resection of the bladder mass. During cystoscopy we found a solid mass approximately 3 cm in size along the right lateral wall of the bladder. Bilateral orifices had clear efflux of urine and there were no other noted lesions. We proceeded with transurethral resection of the mass and histopathologic examination. The hematoxylin and eosin stain revealed neoplastic cells with focal presence of hemosiderin deposition (Figure 1). By immune histochemistry the cancer cells were strongly positive for thyroglobulin (Figure 2), TTF-1 factor, p53 oncoprotein, Leu-7 antigen, E-cadherin and cytokeratins (7, 8/18, 19). Moderate staining was noted for synaptophysin, chromogranin, vimentin, and low expression for epithelial membrane antigen, S-100 protein and cytokeratin 34BE12. There was a negative reaction to calcitonin, CDFP-15, P63, CEA, HMB-45, HHF-35, RCC, CD10, CA-125, SMA, desmin, and hormone receptors for estrogen and progesterone. The above characteristics were more consistent with metastatic follicular thyroid cancer with neuroendocrine differentiation.

The patient's gross hematuria resolved and treatment continued with a course of chemotherapy. Cystoscopy three months postoperatively revealed no recurrence or residual mass.

## 3. Discussion

Most metastatic cases to the urinary bladder are discovered during autopsy reports [2]. It is thought to occur either by extension from retroperitoneal involvement or from venous emboli implantation into the serosa [3]. Vast majority of these patients present with hematuria and/or obstructive urinary symptom [4].

 The most common primary tumors that metastasize to the bladder in the order of most to least common according to organ of origin are malignant melanoma, gastric, breast, kidney, lung, and pancreas. Thyroid cancer with metastasis to the bladder is extremely rare, with an incidence reported <0.5%. Furthermore, primary bladder cancer may coexist with a secondary neoplasm, with a reported incidence of 5.4% [5]. 

Our case involved a patient with metastatic thyroid cancer 5 months after treatment for her primary tumor. To our knowledge, there are only 2 cases in the literature that report metastatic thyroid cancer to the bladder with gross hematuria as first disease symptom [6, 7]. The interesting feature of our case was that the initial presentation of the metastatic disease was a bladder mass combined with gross hematuria. The use of immunohistochemistry methods was very helpful and such methods should be applied in similar cases.
